# Protective Effect of the Chinese Herb Draba Scabra on the Sepsis Myocarditis in Molecular Mechanism

**DOI:** 10.1155/2022/2313813

**Published:** 2022-08-09

**Authors:** Xiaoxin Gao, Rujun Zhang, Fuxing Hu, Qingan Chen, Zhenlin Lei, Yanan Yang, Jia Tian, Ling Huang

**Affiliations:** ^1^Intensive Medical Unit, Hainan General Hospital, Hainan Affiliated Hospital of Hainan Medical University, Haikou, China; ^2^Department of Cardiology, Hainan Province Clinical Medical Center, Hainan General Hospital, Hainan Affiliated Hospital of Hainan Medical University, Haikou, China; ^3^Intensive Medical Unit, Hainan Second People's Hospital, Wuzhishan, China; ^4^Hainan Province Key Laboratory for Drug Preclinical Study of Pharmacology and Toxicology Research, Hainan Medical University, Haikou, China; ^5^School of Hainan Provincial Drug Safety Evaluation Research Center, Hainan Medical University, Haikou, China; ^6^Key Laboratory of Emergency and Trauma of Ministry of Education, Hainan Medical University, Haikou, China; ^7^Research Unit of Island Emergency Medicine, Chinese Academy of Medical Science, Hainan Medical University, Haikou, China

## Abstract

**Objective:**

The aim of this study was to observe the efficacy and mechanism of Draba scabra in sepsis myocarditis.

**Methods:**

The efficacy and pathways of action of Draba scabra on septic myocarditis were evaluated by making a rat model of sepsis with appendix perforation, using Draba scabra for pharmacological intervention, and measuring serum inflammatory factors, cardiac function indexes and parameters, and P38 protein expression in each group of rats, respectively.

**Results:**

The inflammatory factor level, apoptotic index of cardiomyocytes, and P38-MARK protein were significantly higher, while the cardiac function index and hemodynamic index were significantly decreased in group B, while the opposite was true in group E. The treatment was also found to be dose-dependent.

**Conclusion:**

Draba scabra pretreatment effectively reduces the inflammatory response and improves hemodynamic indexes in septic rats. The mechanism may be via the P38-MARK pathway to protect the myocardium.

## 1. Introduction

Sepsis is a life-threatening organ dysfunction caused by dysregulation of the organism's response to infection, and the mortality rate caused by this syndrome is estimated to be 30–50% [1]. Through the use of radionuclide angiography techniques as well as cardiac ultrasound, sepsis has been shown to affect cardiomyocytes. Sepsis myocarditis affects 40–50% of sepsis patients, and heart failure affects 70% sepsis patients. Sepsis-induced cardiac dysfunction is characterized primarily by a significant decrease in cardiac systolic function, ejection fraction, impaired left ventricular diastolic function support, and vascular hyporesponsiveness [2–4]. Most researchers believe that the pathogenesis of sepsis myocarditis is related to the organism's excessive inflammatory response, myocardial mitochondrial and endoplasmic reticulum dysfunction, oxygen radical damage, renin-angiotensin system alteration, and autonomic dysfunction.

With no specific therapeutic agents available, the current treatment for sepsis consists of antibiotics, drainage of inflammatory lesions, and organ function support. Immunotherapeutic drugs such as TNF- and TLR-4, which have been reported in foreign studies, have been used on a small scale in the clinic, bringing new hope for the treatment of sepsis, but no successful efficacy has been achieved thus far [5]. As a result, it is critical to discover therapeutic drugs that inhibit the inflammatory response while improving cardiopulmonary function with pinpoint accuracy and safety.

Many natural drugs have the advantage of having relatively few toxic side effects and unique mechanisms of action, making them important targets for anti-inflammatory drug research and development. Draba scabra, a common Chinese herbal medicine scabra, has been retrieved from de, and it was first described in Shennong Ben Cao Jing. Clinical studies have revealed that Draba scabra has cardiotonic effects, improving cardiac contractility, slowing heart rate, increasing output, and lowering venous pressure in a failing heart, as well as relaxing bronchial smooth muscle, lowering pulmonary vascular resistance, and retracting dilated heart chambers, thereby improving pulmonary edema and heart failure symptoms and signs [6, 7].

Modern pharmacological studies suggest that Draba scabra can lower plasma cyclic adenosine monophosphate (cAMP) concentrations and downregulate myocardial tissue angiotensin II (Ang II), plasma aldosterone (ALD), and myocardial hydroxyproline (Hyp) content [8–10], which could be one of the mechanisms for scapularis' cardioprotective effects. The effects of Draba scabra are mostly focused on the protection of cardiac function in current retrievable clinical and experimental studies, and there are no studies related to the inhibition of the inflammatory response by Draba scabra and foreign literature. As a result, we conducted this study to examine the myocardial cytoprotective effects of different doses of Draba scabra on sepsis myocarditis in rats, to provide a new therapeutic ideas for the prevention of sepsis.

## 2. Materials and Methods

### 2.1. Materials

#### 2.1.1. Animals

Eight-to ten-week-old specific pathogen-free (SPF) grade male Sprague-Dawley (SD) rats were raised in strict accordance with the animal feeding standard procedures. The environmental temperature was maintained at 22–25°C, and the humidity was 45–55%. The rats were ventilated 6–10 times per hour. The water and feed were fed on time every day, and the padding was changed every 2 days. The environmental cleanliness was of grade 100,000, with a colony count < 12.2/plate, noise < 60 dB (A), and light and dark conditions for 12h each. All experimental animals were fasted 12 h before surgery, but water was not prohibited.

#### 2.1.2. Drugs and Reagents

a. Draba scabra granules (Guangdong Party Pharmaceutical Co.); b. ELISA kits (Unitech Biotech); c. Rabbit antiphospho-P38 MAPK (Thr180+Tyr182) antibody (Servicebio).

#### 2.1.3. Methods

- After a week of saline and Draba scabra gavage, the sepsis model was established using the cecal ligation-perforation method. The rats were anesthetized by an intraperitoneal injection of phenobarbital at 30 mg/kg, and the skin was prepared. After the skin was fixed while the rats were placed in the supine position, the skin was disinfected and covered with a towel. A 1-cm long abdominal incision was made along the midline and through the linea alba to enter the abdominal cavity. After cecal exposure, ligation was performed. The cecum was placed back into the abdominal cavity after an 18-needle puncture to cause the intestinal contents to overflow. The abdominal incision was sutured in layers, and saline at 1 ml/100g was injected into the abdominal cavity. The rats with sepsis-compatible manifestations such as mental fatigue, restlessness, chills, abdominal distension, increased secretions out of the corners of their eyes, and piloerection were observed for 24 h after the initiation of the model, and successful modeling was then confirmed. After the sepsis model was successfully established, the rats were randomly divided into groups A, B, C, D, and E with the following grouping and intervention methods (see Table1).:
- Hemodynamic indexes were measured 96 h after surgery, when each group of rats was anesthetized again and fixed on the operating table; the airway was opened and intubated, and respiration parameters were adjusted using a small animal ventilator (respiratory rate of 70 breaths/min, respiratory ratio of 1 : 1) to keep the rats breathing. After exposing the right carotid artery, a catheter was inserted to measure peripheral arterial pressure. The catheter was advanced deeper and inserted into the left ventricle to monitor left ventricular systolic pressure (LVSP), left ventricular end-diastolic pressure (LVEDP), and change rate of left intraventricular pressure (±dP/dtmax). Following the completion of the measurements, the operation was performed on ice, and the heart tissue (ventricle, left ventricle, and aorta) was removed.
- Hematoxylin-eosin staining of cardiac myocytes: The hearts of each group of rats were removed and fixed with 4% paraformaldehyde, and the myocardial tissue was taken from the lateral wall of the left ventricle, dehydrated, embedded, sectioned, dewaxed, and sealed after hematoxylin-eosin staining.
- ELISA to detect inflammatory factors in postsepsis myocardial tissue: After removing the tissue blocks, they were rinsed 2 to 3 times with precooled D-Hanks buffer to remove blood stains, cut into small pieces, and placed in a homogenizer. Add 10 times the tissue volume of the protein extraction reagent and thoroughly homogenize on ice. Shake the homogenate before transferring it to a centrifuge tube 4°C ice bath for 30 minutes to ensure complete lysis, centrifuge at 12000r/min for 5 minutes, collect the supernatant, and detect the content of interleukin-6 (IL-6), tumor necrosis factor-*α* (TNF-*α*), and interleukin-1 (IL-1) by using ELISA kits.
- Detection of P38 protein expression in cardiac myocytes using Western blotting: SDS-PAGE electrophoresis, film transfer, film washing, and antibody incubation were performed after myocardial tissue was withdrawn from the −80°C refrigerator and lysed following quick grinding, protein content was evaluated using the BCA kit method, and then fixed and quantified. Film exposure is followed by development and fixing. After scanning and archiving the film, the photo was organized and decolored in PhotoShop, and the optical density values of the target bands were analyzed by the Alpha software processing system. The primary antibody P38 was employed at a concentration of 1:400, and the secondary antibody was used at 1:3000.

#### 2.1.4. Statistical Methods

For measurement data, the *t*-test was used to compare groups, and for count data, the *X*^2^ or Fisher's exact probability approach was used to compare components. The significance level was set at *p* < 0.05.

## 3. Results

**Figure 1:**
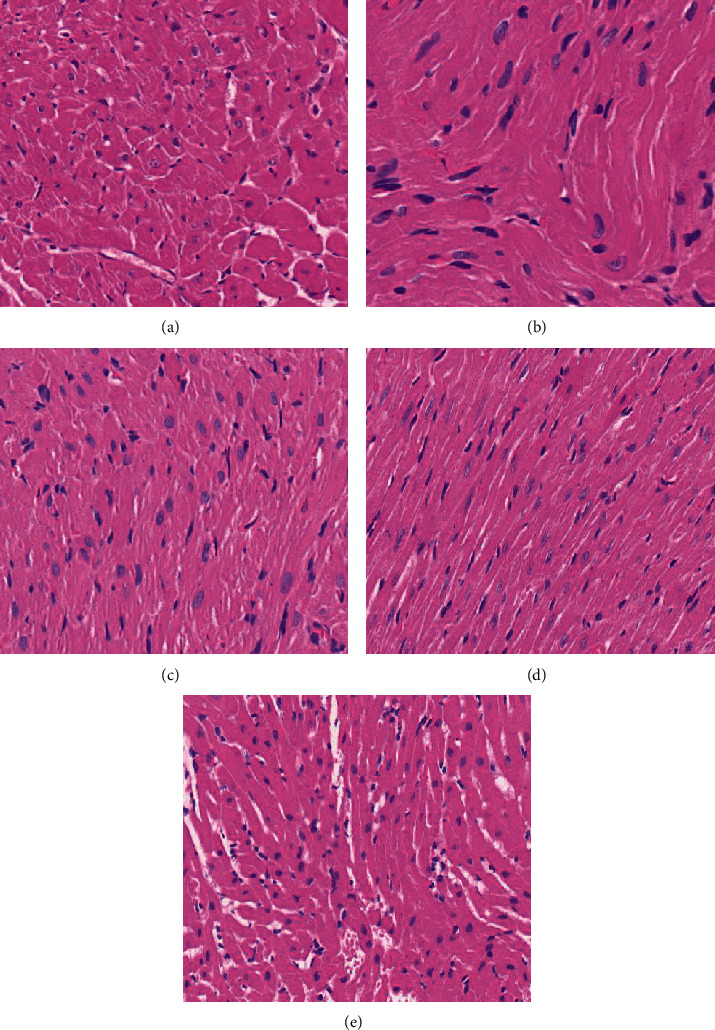
Histopathological manifestations of the myocardium in each group of rats. Note: A is the sham group, B is the sepsis group, C is the low-dose Draba scabra group, D is the medium-dose Draba scabra group, and E is the high-dose Draba scabra group (HEX40).

**Figure 2:**
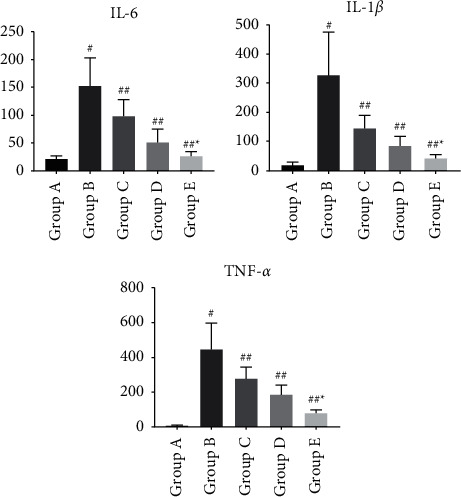
Changes in serum inflammatory factors in each group of rats. Note: comparison of inflammatory factors in the serum of rats in each group, compared with group A ^#^*P* < 0.01; compared with group B ^#^*P* < 0.05; compared with group C ^*∗*^*P* < 0.01.

**Figure 3:**
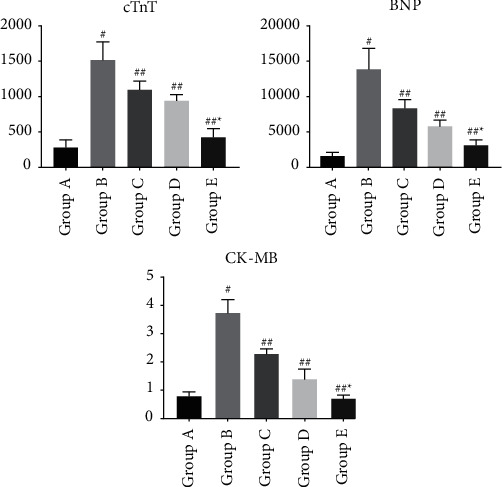
Changes in cardiac function indexes in each group of rats. Note: compared with group A ^#^*P* < 0.01; compared with group B ^#^*P* < 0.05; compared with group C ^*∗*^*P* < 0.01.

**Figure 4:**
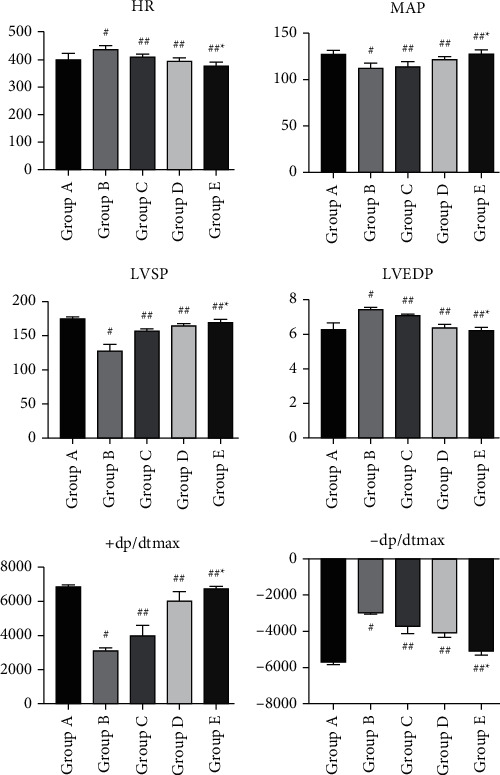
Changes in cardiac function parameters in each group of rats. Note: compared with group A ^#^*P* < 0.01; compared with group B ^#^*P* < 0.05; compared with group C ^*∗*^*P* < 0.01.

**Figure 5:**
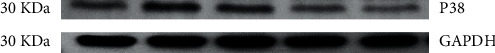
Expression of P38 protein in each group of rats.

- The sepsis ratswere depressed, with reduced drinking, feeding, and activity, erect hair reaction, increased abdominal muscle tone, and dissection of the abdomen revealed cloudy, faecal-smelling abdominal exudate overflow; the intestinal wall of the cecum ligated and punctured section of the intestinal canal was congested and edematous, dull in color, and even purplish-black necrosis.
- Rats in group A had neatly arranged myocardial fibers with no fiber breaks or inflammatory cell infiltration; rats in group B had disorganized myocardial fibers with myocardial cell edema, myocardial fiber breaks, and a large amount of inflammatory cell infiltration; and rats in the C, D, and E groups had significantly reduced myocardial tissue edema, as shown in Figure 1.
- Serum levels of IL-1, TNF-, and IL-6 in rats of each group: Serum levels of IL-1, TNF-*α*, and IL-6 in rats of group B were significantly increased when compared to group A; they were significantly decreased in groups C, D, and E, as shown in Figure 2.
- Troponin-T (cTnT), creatine kinase-MB (CK-MB), and BNP serum levels were significantly higher in group B than in group A and significantly lower in groups C, D, and E, as shown in Figure 3.
- The HR of group B was significantly increased compared to that of group A, but MAP, LVSP, LVEDP, +dp/dtmax, and -dp/dtmax were significantly decreased. Compared with group B, HR decreased, and MAP, LVSP, LVEDP, +dp/dtmax, and -dp/dtmax were significantly increased in groups C, D, and E, as shown in Figure 4.
- Compared with group A, p38 protein expression was significantly increased in group B, but it was significantly decreased in the groups D and E, as shown in Figure 5.

## 4. Discussion

Sepsis is a systemic inflammatory response caused by pathogenic microorganisms and their toxins and is one of the leading causes of death in critically ill patients [11]. The heart is one of the most commonly involved target organs in the course of sepsis, and myocardial and lung injury are the initiating links in the progression of sepsis to multiorgan dysfunction [12]. Inflammatory factor invasion of cardiomyocytes [13], Ca+ overload of cardiomyocytes [14], overactivation of the renin-angiotensin system [15], and impaired energy metabolism [16] have all been implicated in the process of myocardial injury. Among these mechanisms, the inflammatory response is undeniably important. During a systemic inflammatory response storm, the organism can release cytokines that inhibit myocardial function, such as TNF-, IL-1, and IL-6, while the signaling pathways involved include NF-*κ*B (P65, I*κ*B), MAPK-P38, PI3K-AKT, and ERK. To better understand the role of the inflammatory response and related mediators in the process of myocardial injury in sepsis, we used cecal ligation perforation to create a sepsis-induced myocardial injury model in rats and examined the levels of inflammatory factors in the serum of rats with successful modeling. The levels of IL-1, TNF-, and IL-6 in the serum of rats in the sepsis group were significantly higher, and there was more myocardial tissue edema and myocardial fiber breakage.

We discovered that the levels of cTnT, CK-MB, and BNP were significantly higher and inversely correlated with changes in hemodynamic parameters such as MAP, LVSP, LVEDP, and +dp/dtmax in the rat sepsis model. This suggests that during sepsis, there is significant myocardial damage and that the corresponding markers are elevated and secreted in large amounts into the circulatory system, affecting cardiac function.

Draba scabra is a cruciferous scape that is commonly used as a herbal medicine, which has a digitalis-like cardiac glycoside effect and diuretic effect, which can increase cardiac output, can reduce cardiac load, and is an effective drug for the clinical treatment of heart failure, according to modern medical research. Draba scabra reduces myocardial apoptosis by promoting ischemic myocardial revascularization and inhibiting oxidative stress, thereby decreasing the area of myocardial infarction and has a significant inhibitory effect on tissue and organ damage caused by myocardial ischemia-reperfusion [17]. The current study found that Draba scabra can effectively reduce the level of inflammatory factors, reduce the degree of myocardial damage, and improve hemodynamic indexes and apoptosis in septic rats in a dose-dependent manner, implying that it may have anti-inflammatory properties.

MAPKs are a type of serine/threonine kinase that is phosphorylated in a cascade before moving from the cytoplasm to the nucleus to transmit signals and participate in cell differentiation, proliferation, and death. The MAPK family includes extracellular regulated protein kinase (ERK), c-Jun amino-terminal kinase (JNK), and P38. The P38-MAPK family is involved in inflammatory factor production as well as pathophysiological processes [18]. In an isolated heart model, P38-MAPK activation was found to mediate LPS-induced myocardial contractile dysfunction and TNF release, as well as indirectly induce IL-1 and IL-6 production to exacerbate cardiomyocyte injury [18]. We discovered that p38 protein levels were significantly higher in rats with sepsis myocarditis. Therefore, it is hypothesized that Draba scabra effectively inhibits cardiomyocyte necrosis through the P38-MARK pathway.

In conclusion, the development of septic myocarditis is associated with the release of large amounts of inflammatory factors, and Draba scabra can effectively protect the myocardium by inhibiting the P38-MARK pathway, thus improving the degree of myocardial injury.

## Figures and Tables

**Table 1:** Grouping and intervention methods.

Group	Grouping situation	Intervention methods
A	Control group	Saline gavage 1 ml/100 g
B	Sepsis group	Saline gavage 1 ml/100 g
C	Low dose draba scabra group	Draba scabra gavage 20 mg/kg·d
D	Medium dose draba scabra group	Draba scabra gavage 40 mg/kg·d
E	High dose draba scabra group	Draba scabra gavage 60 mg/kg·d

## Data Availability

The data used to support the findings of this study are included within the article.
